# Supplementary material for: Intestine-enriched apolipoprotein b orthologs are required for stem cell progeny differentiation and regeneration in planarians
Source: Nat Commun. 2022 Jul 1;13:3803. doi: 10.1038/s41467-022-31385-2 (PMC9249923; doi:10.1038/s41467-022-31385-2)
Supplement: Supplementary file 1 — Supplementary Information [file 41467_2022_31385_MOESM1_ESM.pdf]

Supplementary Information

Intestine-enriched *apolipoprotein b* orthologs are required for stem cell progeny differentiation and regeneration in planarians

Lily L. Wong[1], Christina G. Bruxvoort[1,2,3,4], Nicholas I. Cejda[1,5], Matthew R. Delaney[1], Jannette Rodriguez Otero[6,7], and David J. Forsthoefel[1,8,*]

[1] Genes and Human Disease Research Program, Oklahoma Medical Research Foundation, Oklahoma City, Oklahoma

[2] Current affiliation: Arthritis and Clinical Immunology Research Program, Oklahoma Medical Research Foundation, Oklahoma City, Oklahoma

[3] Current affiliation: Department of Pathology, University of Oklahoma Health Sciences Center, Oklahoma City, Oklahoma

[4] Current affiliation: Department of Veteran Affairs Medical Center - Research Services, Oklahoma City, Oklahoma

[5] Current affiliation: Center for Biomedical Data Science, Oklahoma Medical Research Foundation, Oklahoma City, Oklahoma

[6] Howard Hughes Medical Institute, Department of Cell and Developmental Biology, University of Illinois at Urbana-Champaign, Urbana, Illinois

[7] Current affiliation: Department of Education, Universidad Interamericana de Puerto Rico, San Juan, Puerto Rico

[8] Department of Cell Biology, University of Oklahoma Health Sciences Center, Oklahoma City, Oklahoma

[*]Correspondence should be addressed to DJF (email: david-forsthoefel@omrf.org)

Supplementary Figures and Figure Legends

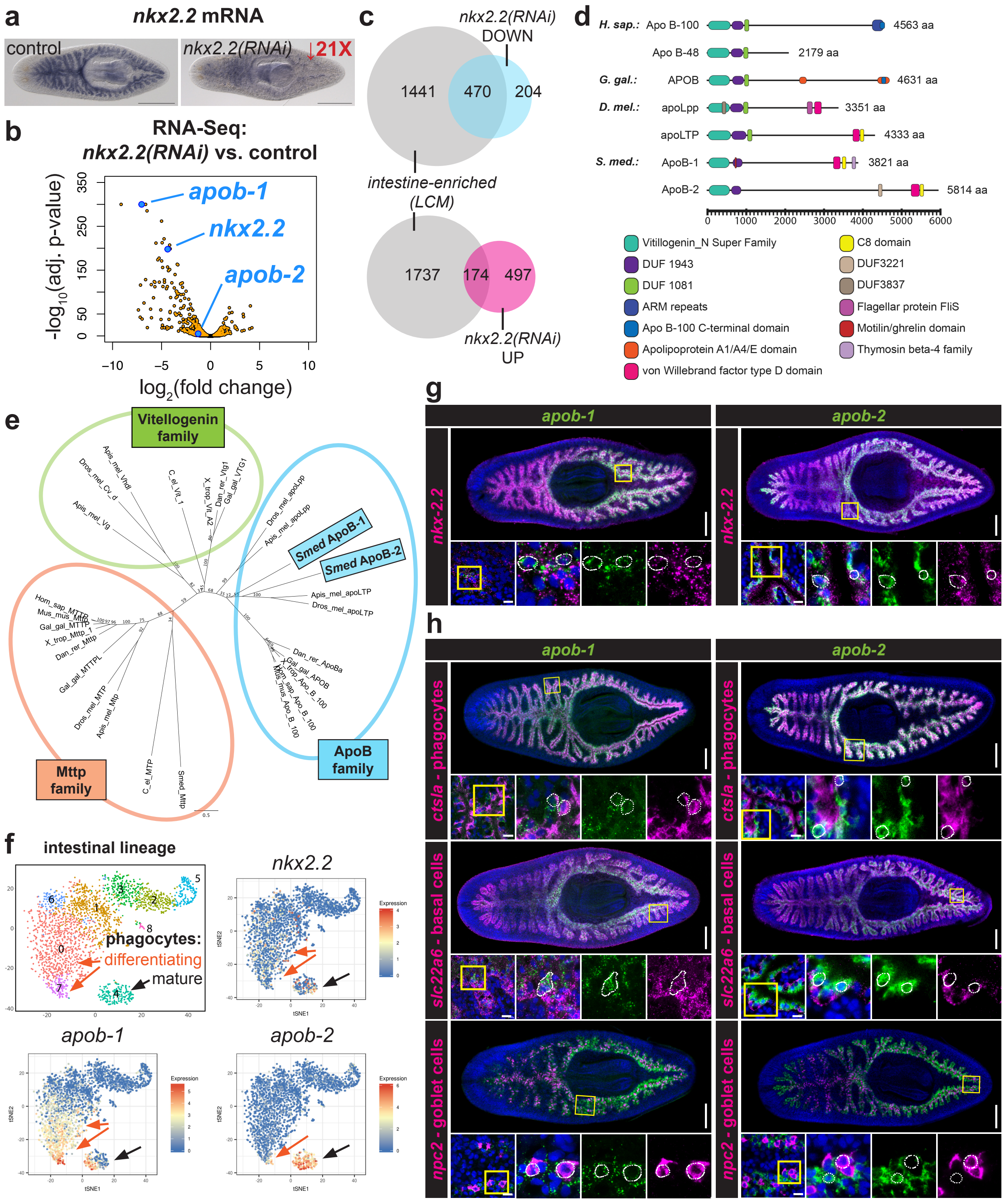

Supplementary Figure 1.

Supplementary Figure 1. *apob-1* and *apob-2* encode intestine-enriched ApoB orthologs. **(a)** Representative *nkx2.2* mRNA in situ expression patterns (blue) in uninjured control (5/5) and *nkx2.2(RNAi)* (6/6) planarians (one experiment). **(b)** Volcano plot showing downregulation of *apob-1, apob-2,* and *nkx2.2* in *nkx2.2(RNAi)* animals. An offset of 1e-300 was added to all FDR-adjusted *p* values to enable plotting of transcripts with *p*=0. n=3 biological replicates per condition in one RNA-Seq experiment. Expression data are provided in Supplementary Data 1. **(c)** 470 downregulated (top) and 174 upregulated (bottom) transcripts in *nkx2.2(RNAi)* animals exhibited intestine enrichment in a previous study[1]. Total numbers of dysregulated transcripts in *nkx2.2(RNAi)* samples were slightly lower than in Supplementary Data 1, because some were undetectable in the intestine data set. **(d)** Conserved domains in human (*H. sap.),* chicken (*G. gal.*), fly (*D. mel.*), and planarian (*S. med.*) ApoB proteins. **(e)** Phylogenetic relationship of planarian (*Smed*) ApoB-1 and ApoB-2 (based on similarity of N-terminal Vitellogenin domains) with closely related protein families in human (*Hom_sap*), mouse (*Mus_mus*), chicken (*Gal_gal*), fly (*Dros_mel*), honeybee (*Apis_mel*), frog (*X_trop*), and *C. elegans* (*C_el*). Branch support is indicated. **(f)** t-SNE plots from single cell transcriptomes[2] showing expression of *nkx2.2, apob-1,* and *apob-2* in the intestinal lineage. All transcripts were enriched in differentiating progeny (subclusters 0/7, orange arrows) and mature phagocytes (subcluster 4, black arrow); *nkx2.2* was also enriched in neoblasts/transition state cells (subcluster 1). **(g)** Double FISH showing co-expression of *apob-1* and *apob-2* (green) with *nkx2.2* mRNA (magenta) in uninjured planarians. Images are representative of 9/9 (*apob-1/nkx2.2*) and 11/11 (*apob-2/nkx2.2*) uninjured planarians from two independent experiments. **(h)** Double FISH showing expression of *apob-1* and *apob-2* (green) in phagocytes and basal cells (magenta) (*apob-1* only), but not goblet cells (magenta). Images are representative of labeling in 8/8 (*apob-1/ctsla*), 11/11 (*apob-2/ctsla*), 13/13 (*apob-1/scl22a6*), 11/11 (*apob-2/slc22a6*), 8/8 (*apob-1/npc2*), and 6/6 (*apob-2/npc2*) uninjured planarians from two independent experiments. In **(g-h),** confocal maximum intensity projections are shown; yellow boxes indicate regions magnified in insets; insets show only a subset of focal planes in low magnification images. Scale bars: 500 µm **(a)**; 200 µm **(g-h)**; 10 µm **(g-h** insets, bottom left**).**

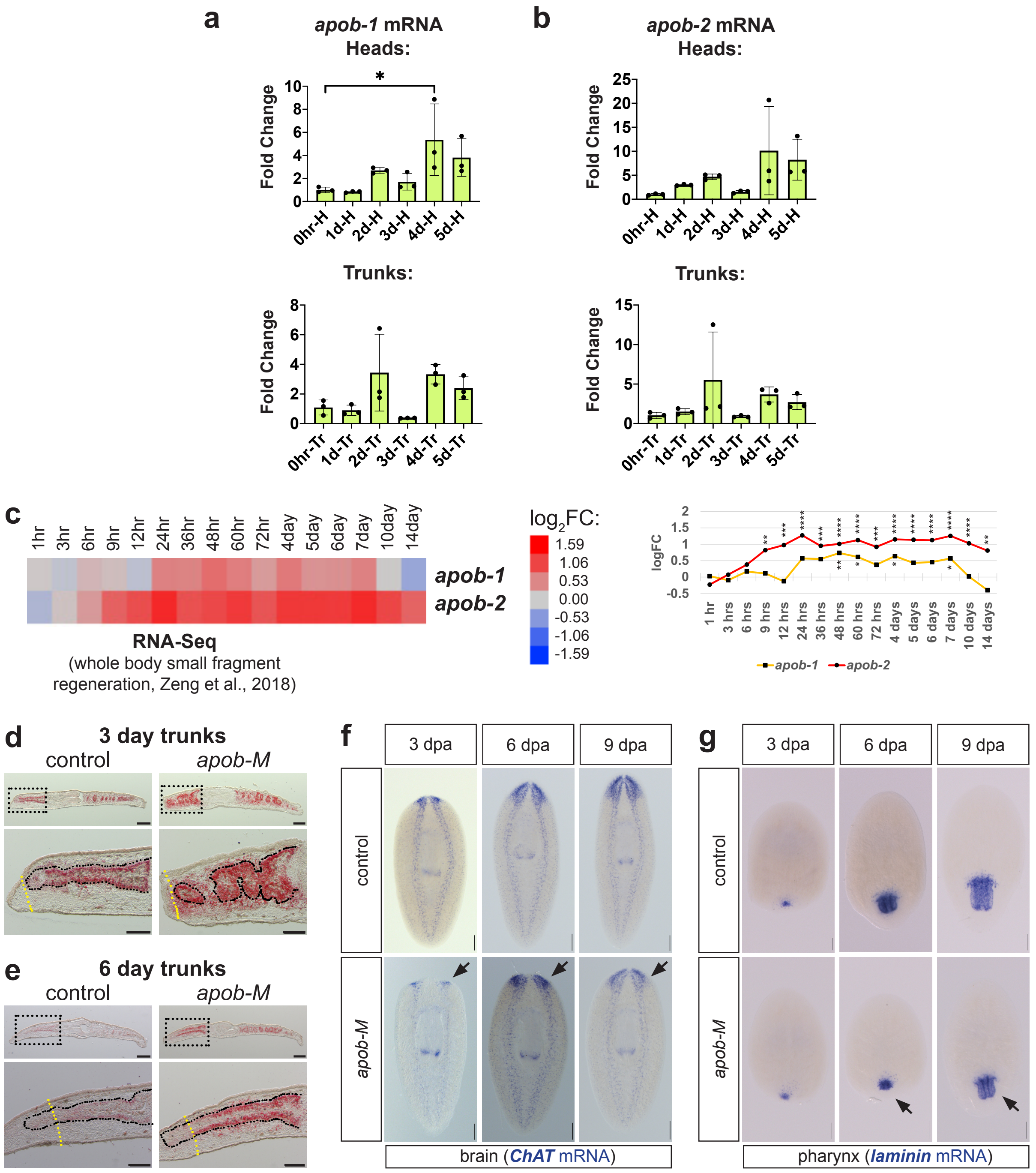

Supplementary Figure 2.

Supplementary Figure 2. Further characterization of *apob* expression and RNAi phenotypes. (a) *apob-1* mRNA trended upwards in head and trunk regenerates (qRT-PCR) (one experiment, n=3 biological replicates per time point). One-way ANOVA (comparison to 0 hr) with Dunnett's T3 multiple comparisons test. Error bars = mean ± S.D. Asterisk (*p=0.014) indicates significant upregulation in 4 dpa heads. **(b)** *apob-2* mRNA trended upwards in head and trunk regenerates (qRT-PCR) (one experiment, n=3 biological replicates per time point). One-way ANOVA (comparison to 0 hr) with Dunnett's T3 multiple comparisons test. **(c)** *apob-1* and *apob-2* mRNA levels were upregulated in whole fragment regeneration RNA-Seq data[3]. Asterisks on the line plot (right) indicate significant $\log_2$ fold changes. FDR-adjusted p values: *p<.05; **p<.01; ***p<.001; ****p<.0001; likelihood ratio test in EdgeR (n=4 biological replicates/time point); exact p values in Supplementary Data 7. **(d-e)** Neutral lipids (red) accumulate in *apob-M* ("mild") regenerates (amputated 7 days after the last dsRNA feeding; days after amputation indicated). Yellow dashed line indicates approximate plane of amputation; black dashes outline intestine. Anterior is left. Representative of one experiment with n=2 animals per condition (>10 sections/animal) at each time point. **(f-g)** Brain (**g**, *ChAT* mRNA ISH, blue) and pharynx (**h**, *laminin* mRNA ISH, blue) regeneration were delayed, but not blocked, by *apob* RNAi. Arrows indicate smaller organs in *apob-M* animals relative to controls (representative images). Images are representative of one experiment with 6/6 fragments for all conditions except 3 day control *ChAT* (7/7), 6 day *apob-M laminin* (5/6), and 9 day control *laminin* (5/5). Scale bars: 200 µm **(d-e,** upper panels**)**; 100 µm **(d-e,** lower panels**)**; 100 µm **(f-g)**.

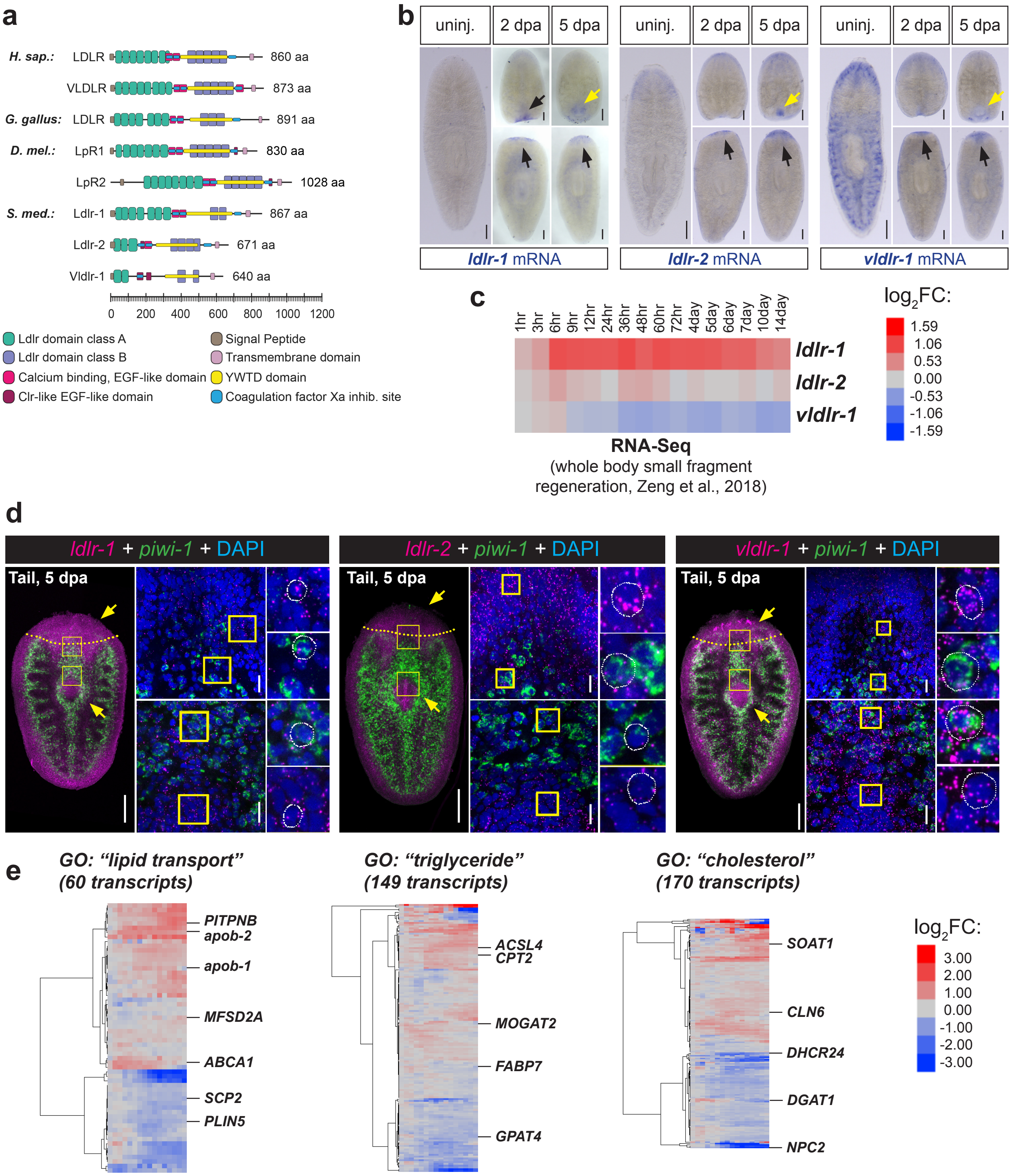

Supplementary Figure 3.

Supplementary Figure 3. Lipoprotein receptor expression is upregulated during planarian regeneration. (a) Schematic of domains in LDLR and related homologs. Only domains common to Ldlr-like proteins in multiple species are shown. Clr-like EGF-like domains are shown only if they do not overlap with Ca-binding EGF-like domains. **(b)** WISH showing upregulation of planarian *ldlr* homologs (mRNA ISH, blue) in blastemas (black arrows) at 2 and 5 dpa, and developing pharynges (yellow arrows) at 5 dpa. Expression is lower in pharynges in uninjured animals and in the pre-existing pharynx in trunk fragments, suggesting downregulation when regeneration is complete. Images are representative of one experiment with 9/9 animals (*ldlr-1* and *ldlr-2* uninjured), 6/6 fragments (2 dpa and 5 dpa *ldlr-2* trunks), 6/8 fragments (2 dpa *ldlr-1* heads), 5/6 fragments (5 dpa *ldlr-2* trunks), or 7/7 animals/fragments (all others). **(c)** *ldlr-1* and *ldlr-2* mRNA levels were upregulated in whole fragment regeneration RNA-Seq data[3]. **(d)** Confocal maximum intensity projections showing that *ldlr* homologs (magenta, mRNA FISH) were expressed in *piwi-1*+ neoblasts (green, mRNA FISH) as well as differentiating (*piwi-1*-negative*)* cells in brain and pharynx (arrows) in 5-day regenerates. Images are representative of one experiment with 7/7 (*ldlr-1* and *vldlr-1*) or 5/5 (*ldlr-2*) regenerates. Yellow boxes indicate regions magnified in insets; insets show only a subset of focal planes in low magnification images. Expression in pre-existing tissue relative to the blastema is higher in FISH samples as compared to WISH samples (Supplementary Fig. 3b), likely due to more rapid completion of the tyramide signal amplification reaction relative to colorimetric development. **(e)** Heat maps showing numerous planarian transcripts related to lipid metabolism that were up- and down-regulated during regeneration in whole fragment regeneration RNA-Seq data (Zeng et al., 2018). See Supplementary Data 3 for transcript IDs and expression data. Scale bars: 200 µm **(b)**; 200 µm **(d,** left panels**)**; 20 µm **(d,** middle panels**)**.

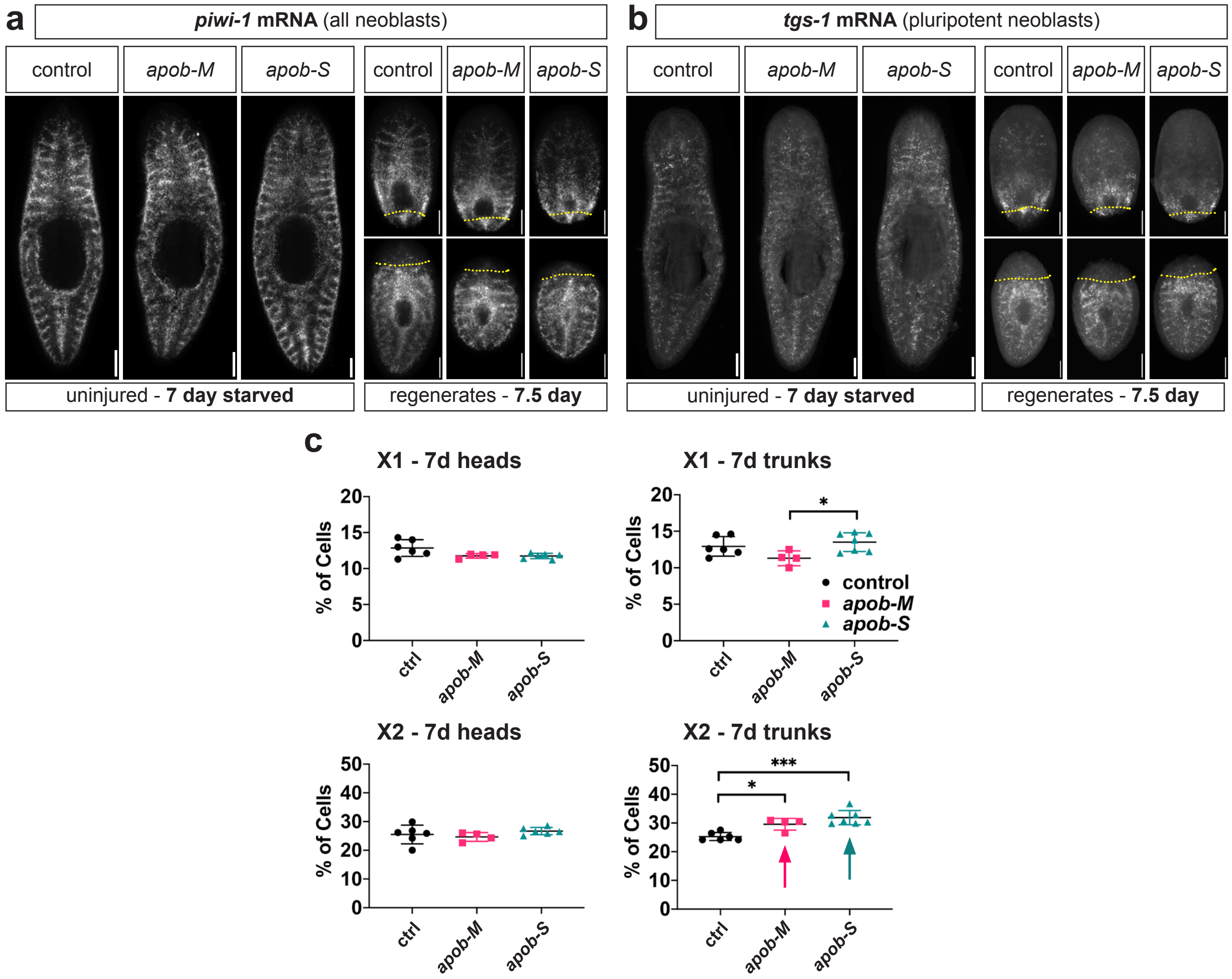

Supplementary Figure 4.

Supplementary Figure 4. Further characterization of effects of *apob* inhibition on neoblasts and neoblast progeny. (a) Distribution of *piwi-1*-expressing neoblasts (gray) in uninjured (7 day starved, left panels) animals and 7.5 dpa head and tail regenerates (right panels). Control, *apob-M (*“mild”), and *apob-S* (“severe”) conditions are indicated. Whole mount FISH; representative images from one experiment: control (10/10 uninjured, 7/7 heads, 6/6 tails); *apob-M* (8/8 uninjured, 6/6 heads, 5/5 tails); *apob-S* (5/5 uninjured, 5/5 heads, 6/6 tails). **(b)** Distribution of *tgs-1*-expressing neoblasts (gray) in uninjured (7 day starved, left panels) animals and 7.5 dpa head and tail regenerates (right panels). Whole mount FISH; representative images from one experiment: control (10/10 uninjured, 6/6 heads, 6/6 tails); *apob-M* (11/11 uninjured, 5/5 heads, 5/5 tails); *apob-S* (5/5 uninjured, 6/6 heads, 4/4 tails). Dotted lines, approximate amputation plane **(a,b)**. **(c)** Percentage of cells in X1 and X2 in 7 day head (left) and trunk (right) regenerates. Arrows indicate significant increases in X2: *p=0.034 (X1, 7d trunks); *p=0.016 (X2, 7d trunks); ***p=0.0001 (X2, 7d trunks). n=6 (control and *apob-S*), n=4 (*apob-M*) biological replicates from one experiment. One-way ANOVA with Dunnett's (X1, 7 day heads) or Tukey’s (all others) multiple comparisons test. Error bars = mean ± S.D. Scale bars: 200 μm **(a-b)**.

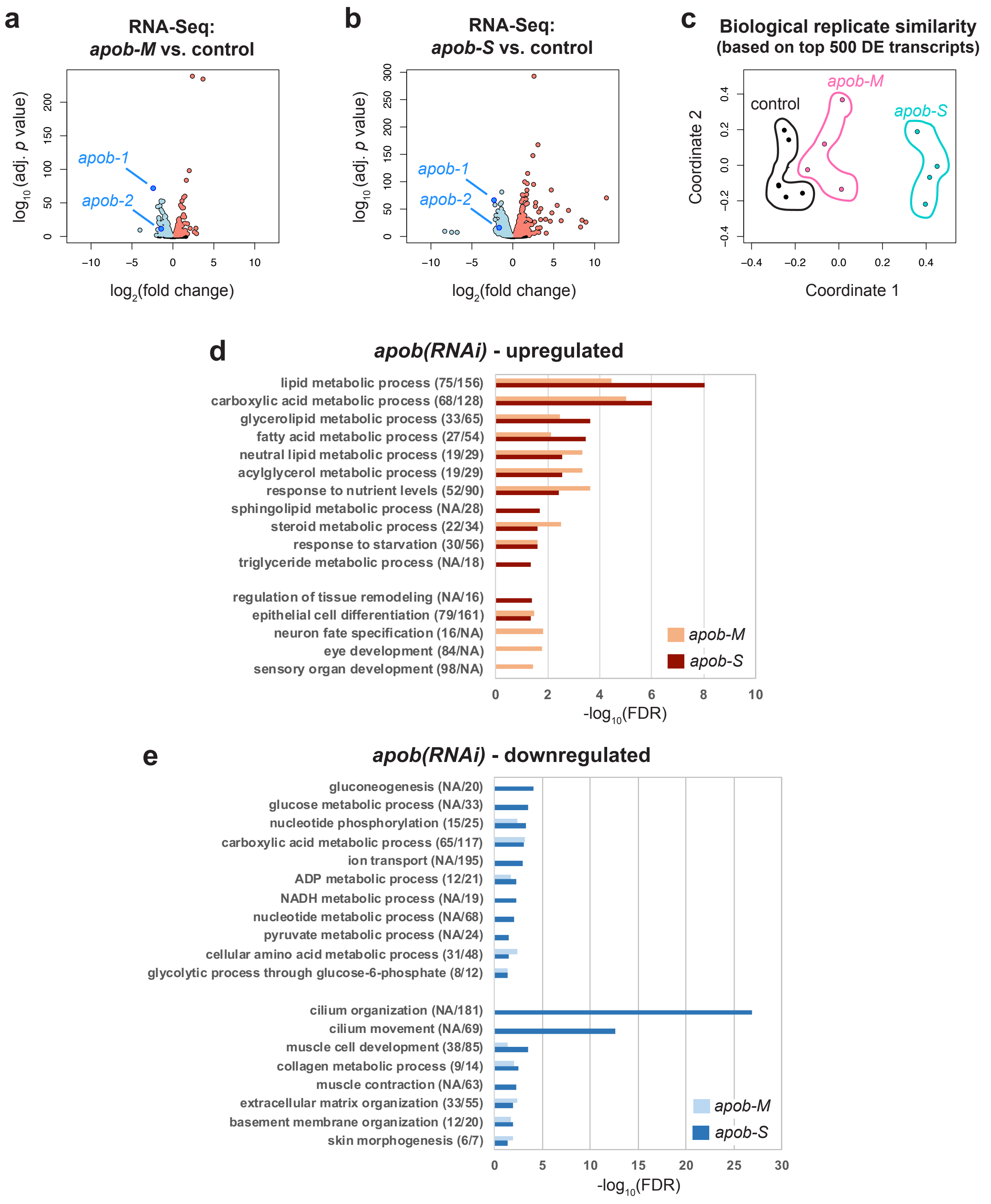

Supplementary Figure 5.

Supplementary Figure 5. *apob* RNAi dysregulates transcripts involved in metabolism- and differentiation-related processes. (a-b) Volcano plots showing significantly downregulated (blue) and upregulated (pink) transcripts in *apob-M* ("mild") **(a)** and *apob-S* ("severe") **(b)** uninjured animals. n=6 (control), n=4 (*apob-M),* or n=4 (*apob-S)* biological replicates. Expression data are provided in Supplementary Data 5. **(c)** Multi-dimensional scaling plot showing similarity of control and RNAi sample libraries, using the biological coefficient of variation method to calculate distances between each library based on the 500 most variable transcripts across all samples. **(d-e)** Gene Ontology Biological Process categories over-represented among transcripts upregulated **(d)** or downregulated **(e)** by *apob* RNAi. Numbers of transcripts dysregulated indicated in parentheses (*apob-M/apob-S*). NA, not applicable (GO category not enriched in *apob-M* or *apob-S*). $-\log_{10}$(FDR) on X-axis.

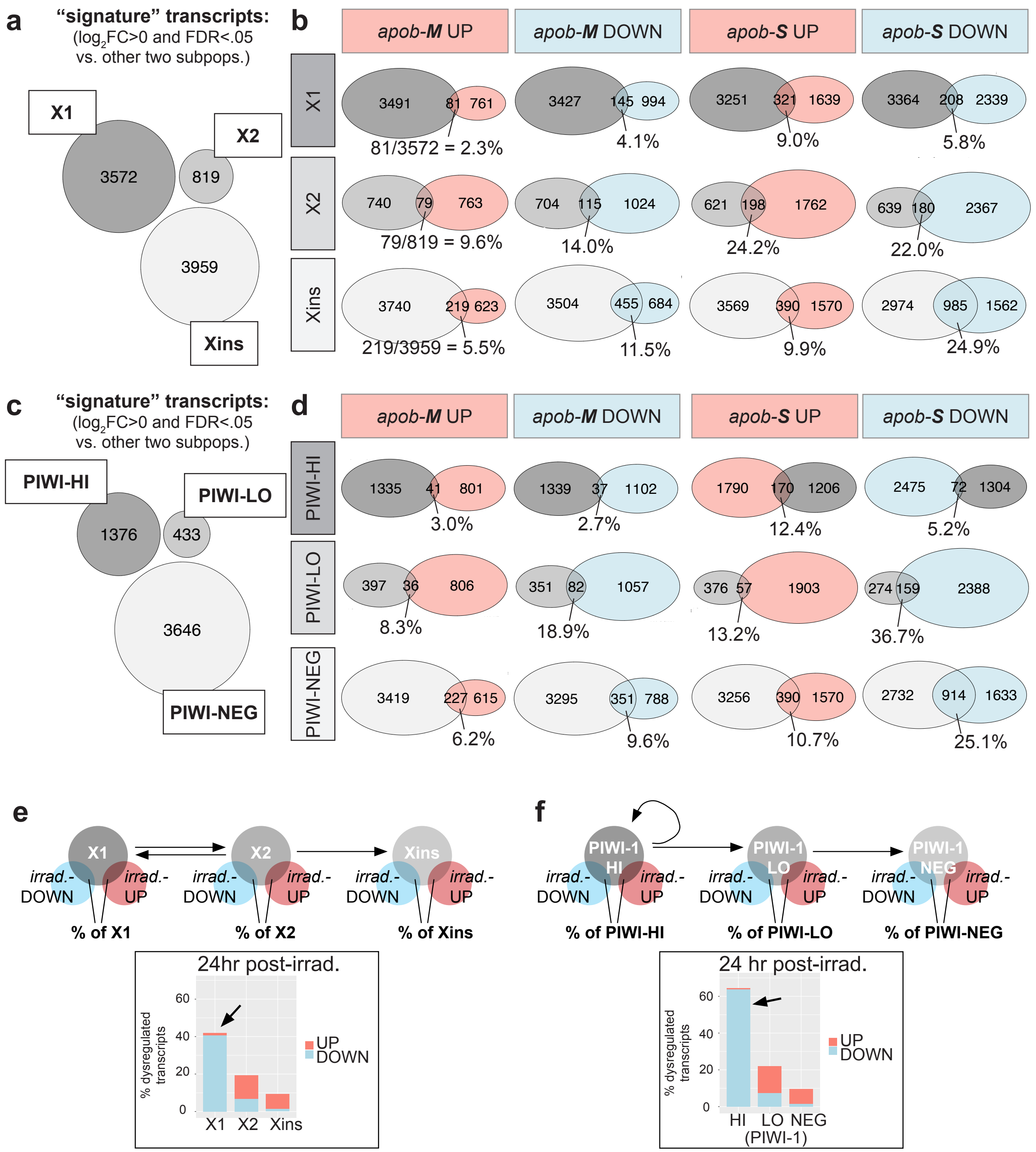

Supplementary Figure 6.

Supplementary Figure 6. *apob* RNAi preferentially dysregulates transcripts in differentiating neoblast progeny and mature post-mitotic cells. (a) Venn diagram showing no overlap between X1, X2, and Xins "signature" transcripts (defined as having logFC>0 (FDR<.05) vs. transcripts in both other fractions). **(b)** Venn diagrams showing overlap between X1, X2, and Xins signature transcripts and transcripts up or down in *apob-M* ("mild") and *apob-S* ("severe") planarians. Percentages of X1/X2/Xins dysregulated are indicated. **(c)** Venn diagram showing no overlap between PIWI-HI, PIWI-LO, and PIWI-NEG signature transcripts (defined as having logFC>0 (FDR<.05) vs. transcripts in both other fractions). **(d)** Venn diagrams showing overlap between PIWI-HI, PIWI-LO, and PIWI-NEG signature transcripts and transcripts up or down in *apob-M* and *apob-S*. Percentages of PIWI-HI/PIWI-LO/PIWI-NEG dysregulated are indicated. **(e-f)** X1-enriched **(e)** and PIWI-HI-enriched **(f)** signature transcripts were preferentially downregulated in planarians 24 hr post-irradiation. Venn diagrams at top show analysis schemes: percent of X1/X2/Xins **(e)** and PIWI-HI/PIWI-LO/PIWI-NEG **(f)** signature transcripts that overlap with transcripts dysregulated in planarians 24 hr post-irradiation. Histograms show percentage of signature transcripts up- (red) and down-regulated (blue) in planarians 24 hr post-irradiation. Analysis of data from [3] (see Methods).

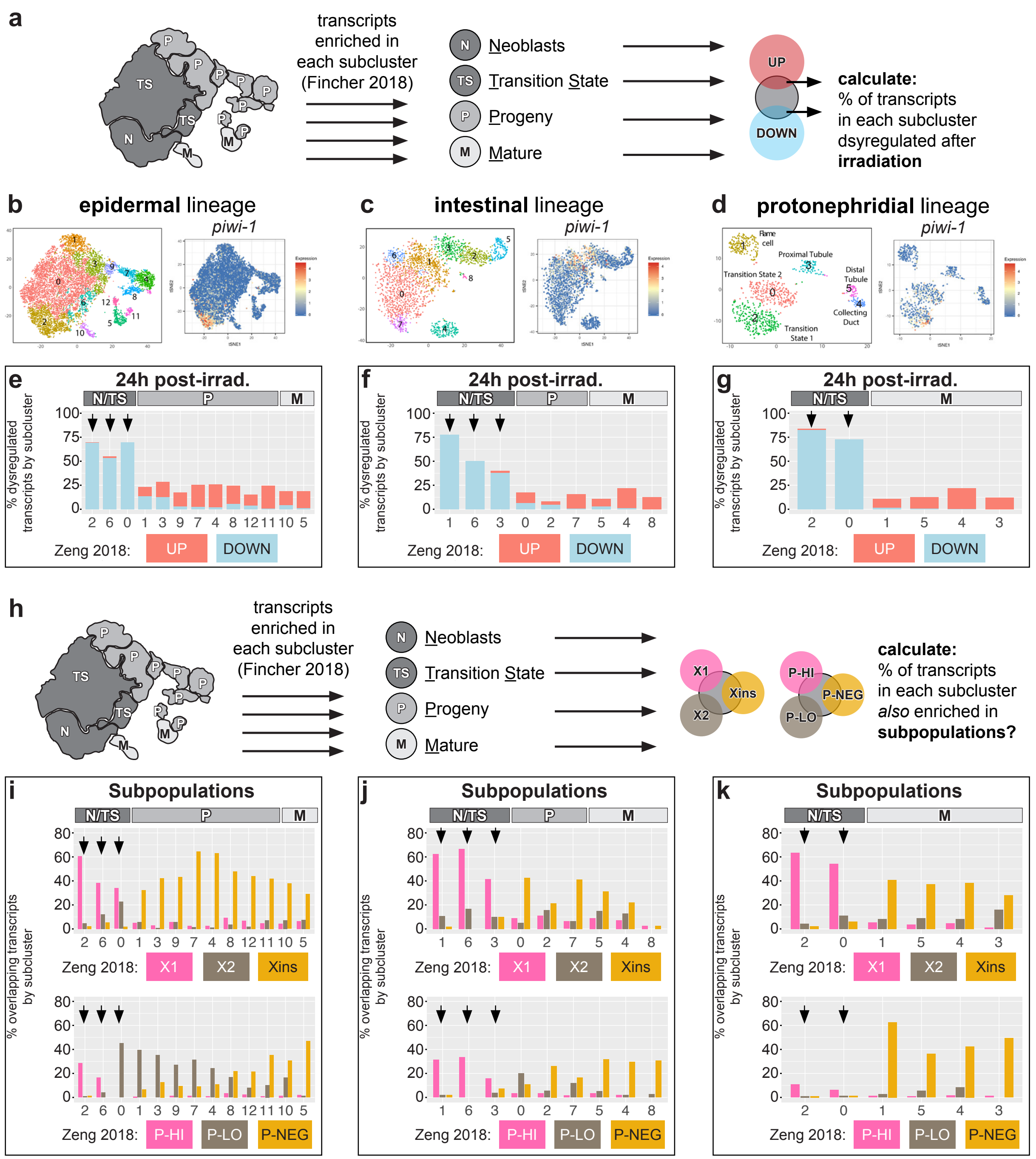

Supplementary Figure 7.

Supplementary Figure 7. Mapping of transcripts from 24 hr post-irradiation and cell-state-enriched subpopulations to single-cell subclusters in three lineages. (a) Schematic example (for epidermal lineage) illustrating how transcripts dysregulated in 24 hr post-irradiation animals[3] were cross-referenced with neoblast (N), transition state (TS), progeny (P), and mature (M) cell state subclusters[2]. See Methods for details. **(b-d)** t-SNE plots (digiworm.wi.mit.edu) indicate subclusters and *piwi-1* mRNA expression for each lineage. **(e-g)** Cross-referencing of scRNA-Seq data[2] with RNA-Seq data from whole planarians 24 hr post-irradiation[3]. Histograms showing that irradiation preferentially caused downregulation of transcripts enriched in neoblast ("N") and transition state ("TS") subclusters in multiple cell type lineages (arrows), by contrast to the effects of *apob* RNAi (see also Fig. 5). **(h)** Cell state schematic and Venn diagrams show analysis strategy to calculate proportion of subcluster-enriched transcripts[2] also enriched in sorted planarian cell subpopulations[3]. **(i-k)** Cross-referencing of scRNA-Seq data[2] with bulk sorted cell RNA-Seq data[3]. Histograms showing that X1/PIWI-HI ("P-HI") signature transcripts were primarily enriched in neoblast ("N") and transition state ("TS") subclusters, and that Xins/PIWI-NEG ("P-NEG") signature transcripts were enriched in progeny ("P") and mature ("M") cell state subclusters. In epidermal and intestinal lineages, X2/PIWI-LOW ("P-LO") signature transcripts were most highly enriched in neoblast/transition state and progeny subclusters. In the protonephridial lineage X2/PIWI-LO transcripts were more uniformly distributed, possibly due to fewer subclusters (and/or lower resolution of transition states) in this scRNA-Seq dataset.

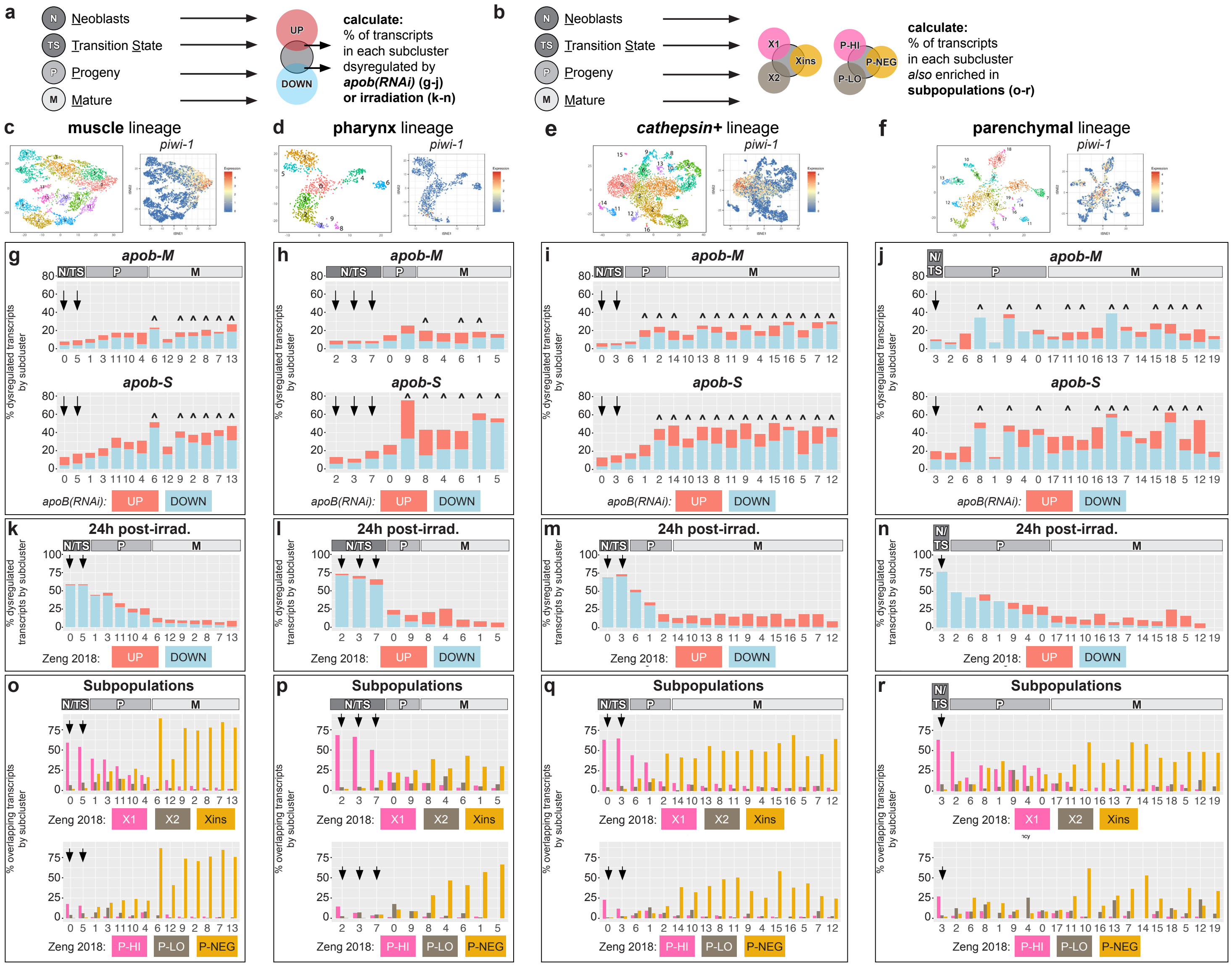

Supplementary Figure 8.

Supplementary Figure 8. Additional examples of dysregulation of transcripts in differentiating neoblast progeny and mature cells by *apob* RNAi. (a) Generic scheme used to identify overlap of transcripts enriched in specific subclusters/cell states[2] that were dysregulated in *apob(RNAi)* planarians (this study) or 24 hr post-irradiation[3]. **(b)** Generic scheme to calculate proportion of cell-state-enriched transcripts[2] also enriched in sorted planarian cell subpopulations[3]. **(c-f)** t-SNE plots (digiworm.wi.mit.edu) indicate subclusters and *piwi-1* mRNA expression for muscle, pharynx, *cathepsin+,* and parenchymal lineages. **(g-j)** *apob* knockdown dysregulated greater proportions of transcripts in progeny ("P") and mature ("M") subclusters in multiple cell type lineages. Arrows indicate less-affected transcripts in neoblast/transition state ("N/TS") subclusters. *apob-M* ("mild") and *apob-S* ("severe") conditions are indicated. **(k-n)** Cross-referencing of scRNA-Seq data[2] with RNA-Seq data from whole planarians 24 hr post-irradiation[3]. Transcripts enriched in neoblasts/transition state subclusters were preferentially downregulated 24 hr post-irradiation (arrows), by contrast to the effects of *apob* RNAi **(g-j)**. **(o-r)** Cross-referencing of scRNA-Seq data[2] with bulk sorted cell RNA-Seq data[3]. Histograms showing that X1/PIWI-HI ("P-HI") signature transcripts were primarily enriched in neoblast/transition state ("N/TS"), and progeny ("P") subclusters; that X2/PIWI-LOW ("P-LO") signature transcripts were most highly enriched in progeny subclusters; and that Xins/PIWI-NEG ("P-NEG") signature transcripts were enriched in mature ("M") cell state subclusters. Carets (^) indicate significant gene expression overlap (**g-j**, $p<0.05$, Fisher's exact test, see Source Data for individual p values).

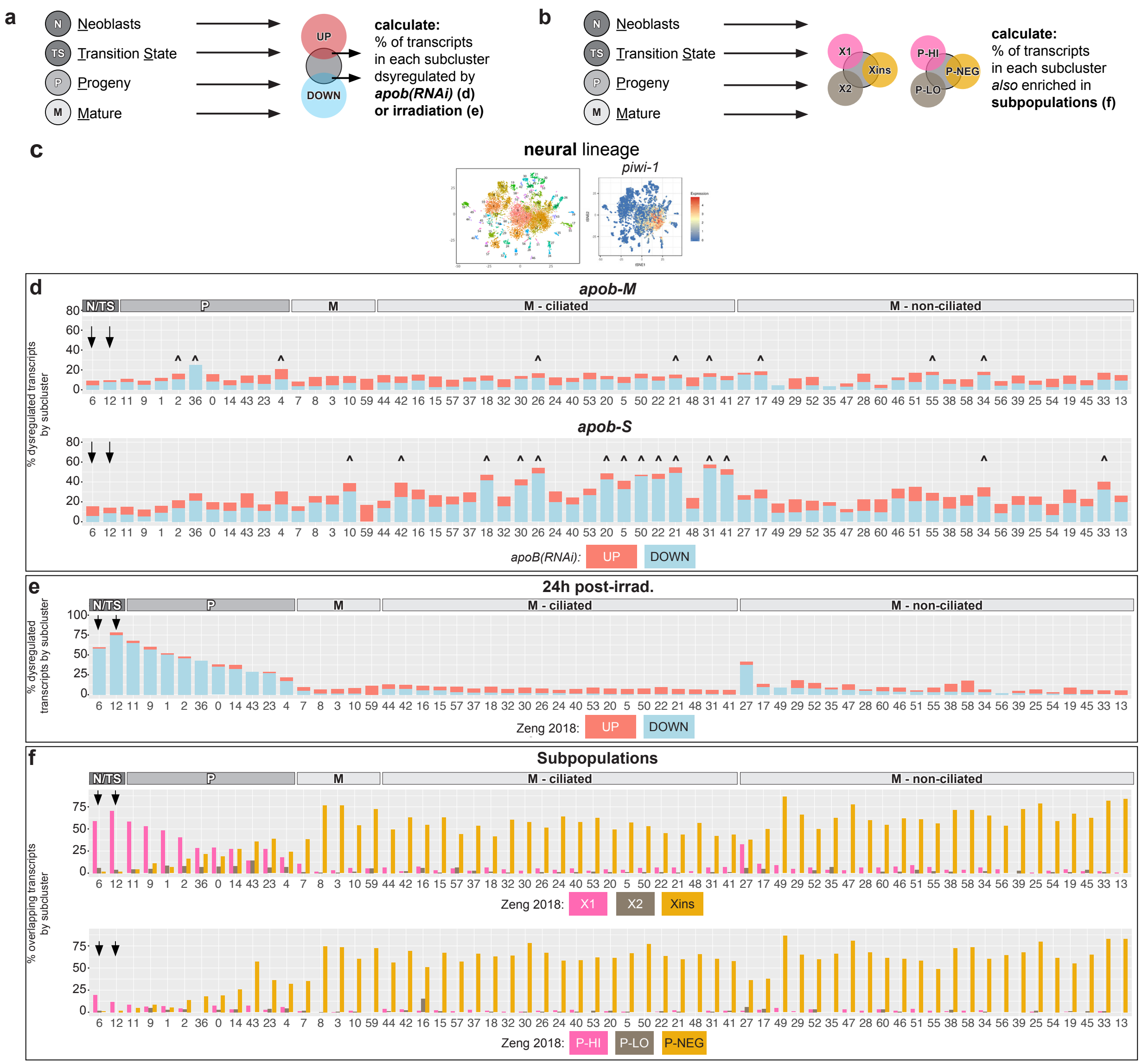

Supplementary Figure 9.

Supplementary Figure 9. Dysregulation of transcripts in differentiating neoblast progeny and mature cells (neural lineage) by *apob* RNAi. (a) Generic scheme used to identify overlap of transcripts enriched in specific subclusters/cell states[2] that were dysregulated in *apob(RNAi)* planarians (this study) or 24 hr post-irradiation[3]. **(b)** Generic scheme to calculate proportion of cell-state-enriched transcripts[2] also enriched in sorted planarian cell subpopulations[3]. **(c)** t-SNE plots (digiworm.wi.mit.edu) indicate subclusters and *piwi-1* mRNA expression for the neural lineage. **(d)** *apob* knockdown dysregulated greater proportions of transcripts in progeny (“P”) and mature (“M”) subclusters in multiple cell type lineages. Arrows indicate less-affected transcripts in neoblast/transition state (“N/TS”) subclusters. *apob-M* (“mild”) and *apob-S* (“severe”) conditions are indicated. **(e)** Cross-referencing of scRNA-Seq data[2] with RNA-Seq data from whole planarians 24 hr post-irradiation[3]. Transcripts enriched in neoblasts/transition state subclusters were preferentially downregulated 24 hr post-irradiation (arrows), by contrast to the effects of *apob* RNAi **(d)**. **(f)** Cross-referencing of scRNA-Seq data[2] with bulk sorted cell RNA-Seq data[3]. Histograms showing that X1/PIWI-HI (“P-HI”) signature transcripts were primarily enriched in neoblast/transition state (“N/TS”), and progeny (“P”) subclusters; that X2/PIWI-LOW ("P-LO") signature transcripts were most highly enriched in progeny subclusters; and that Xins/PIWI-NEG (“P-NEG”) signature transcripts were enriched in mature (“M”) cell state subclusters. Carets (^) indicate significant gene expression overlap (**d**, $p<0.05$, Fisher's exact test, see Source Data for individual *p* values).

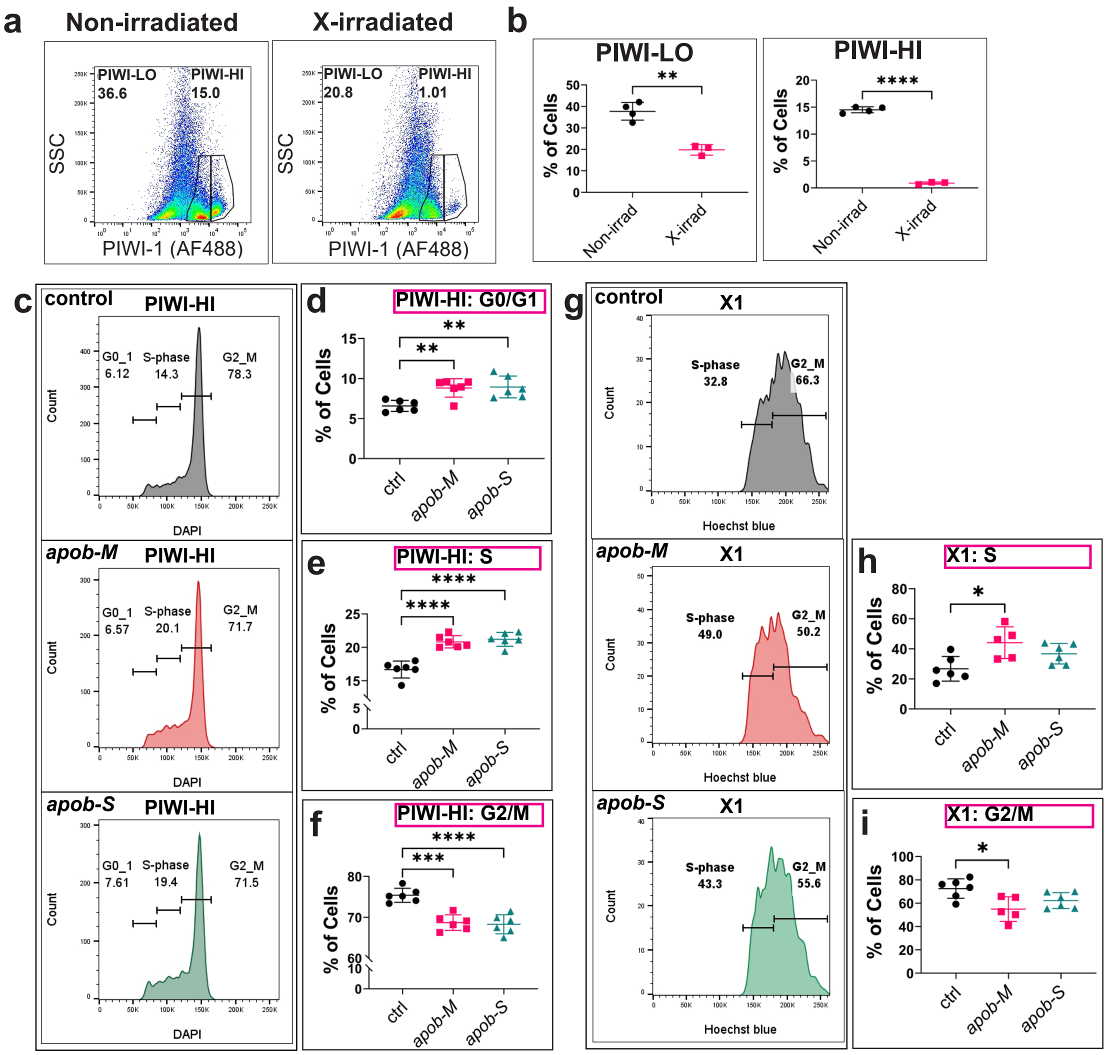

Supplementary Figure 10.

Supplementary Figure 10. PIWI-HI cell population is irradiation sensitive and has an altered cell cycle profile in *apob(RNAi)* animals. (a) Examples of flow dot plots from non-irradiated and 24 hours post irradiated (hpi) planarians showing the dramatic reduction of PIWI-HI cell fraction after irradiation. **(b)** Percentages of PIWI-LO and PIWI-HI cell fractions 24 hpi. PIWI-LO cell population was significantly decreased and PIWI-HI cell population was essentially eliminated, validating the specificity of the custom PIWI-1 antibody. Two-tailed unpaired *t*-test: $^{**}p=0.0012$; $^{****}p<0.0001$. Error bars: mean ± S.D., n=4 (control) or n=3 (X-irradiated) (one experiment). **(c-f)** Gating scheme **(c)** and quantification of the three cell cycle phases (G0/G1, S, G2/M) of the PIWI-HI cell fraction in uninjured animals. *apob(RNAi)* animals had significantly higher proportions of cells in G0/G1 **(d)** and S **(e)** phases, and correspondingly reduced cell fractions in G2/M **(f)** phase. One-way ANOVA with Tukey's multiple comparison test: $^{**}p=0.0083$ (control vs. *apob-M*); $^{**}p=0.0057$ (control vs. *apob-S*); $^{***}p=0.0001$; $^{****}p<0.0001$. Error bars: mean ± S.D., n=6 biological replicates per condition; representative of four independent experiments. **(d-f)**. **(g-i)** Quantification of S and G2/M cell fractions of the X1 subpopulation (>2C DNA content) in uninjured animals. The S phase fraction increased significantly in *apob-M* animals **(h),** while the G2/M fraction decreased in *apob-M* animals **(i)**. One-way ANOVA with Tukey's multiple comparison test: $^{*}p=0.0117$ (X1-S); $^{**}p=0.0115$ (X1-G2/M). Error bars: mean ± S.D., n=5 (*apob-M*) or n=6 (control and *apob-S*) biological replicates; representative of three independent experiments. **(h, i)**.

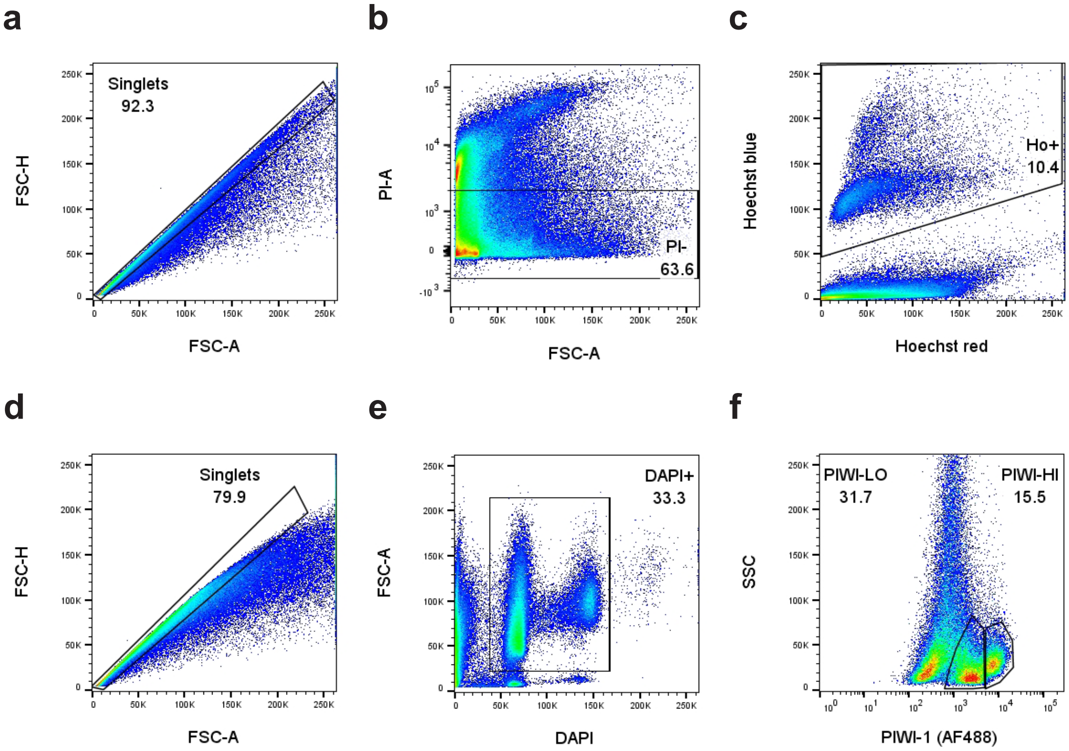

Supplementary Figure 11.

Supplementary Figure 11. Gating strategy for flow cytometry experiments. (a-c) Gating strategy for live cell labeling. **(a)** Forward scatter height (FSC-H) vs. forward scatter area (FSC-A) gate to limit to singlet events. **(b)** Propidium iodide (PI-A) vs. forward scatter area (FSC-A) gate to limit to PI negative (e.g., non-dead) events. **(c)** Hoechst 33342 blue (y-axis) vs. Hoechst 33342 red (x-axis) gates to limit to Hoechst-positive (e.g., ≥2C DNA content) events. Percentages of events after each gating step are indicated. **(d-f)** Gating strategy for PIWI-1 antibody labeling. **(d)** Forward scatter height (FSC-H) vs. forward scatter area (FSC-A) gate to limit to singlet events. **(e)** FSC-A vs DAPI to limit to DAPI-positive events (i.e., 2C-4C DNA content) and exclude debris. **(f)** Side scatter area (SSC) vs Alexa 488 (PIWI-1 antibody labeling) to gate PIWI-LO and PIWI-HI events. Percentages of events after each gating step are indicated.

Supplementary References

1 Forsthoefel, D. J., Cejda, N. I., Khan, U. W. & Newmark, P. A. Cell-type diversity and regionalized gene expression in the planarian intestine. *Elife* **9**, doi:10.7554/eLife.52613 (2020).

2 Fincher, C. T., Wurtzel, O., de Hoog, T., Kravarik, K. M. & Reddien, P. W. Cell type transcriptome atlas for the planarian *Schmidtea mediterranea*. *Science* **360**, doi:10.1126/science.aaq1736 (2018).

3 Zeng, A. *et al.* Prospectively Isolated Tetraspanin(+) Neoblasts Are Adult Pluripotent Stem Cells Underlying Planaria Regeneration. *Cell* **173**, 1593-1608 e1520, doi:10.1016/j.cell.2018.05.006 (2018).
