# Supplementary material for: Intestine-enriched apolipoprotein b orthologs are required for stem cell progeny differentiation and regeneration in planarians
Source: Nat Commun. 2022 Jul 1;13:3803. doi: 10.1038/s41467-022-31385-2 (PMC9249923; doi:10.1038/s41467-022-31385-2)
Supplement: Supplementary file 3 — Description of Additional Supplementary Files [file 41467_2022_31385_MOESM3_ESM.pdf]

Descriptions of Additional Supplementary data Files

Supplementary Data 1. Up- and downregulated transcripts in nkx2.2(RNAi) planarians.

Supplementary Data 2. Gene Ontology Biological Process terms enriched for transcripts up- and downregulated by nkx2.2 RNAi.

Supplementary Data 3. Transcripts annotated with lipid-related Gene Ontology terms in whole fragment planarian regeneration transcriptome.

Supplementary Data 4. Up- and downregulated transcripts in apob(RNAi) "mild" and "severe" planarians.

Supplementary Data 5. Gene Ontology Biological Process terms enriched for transcripts up- and downregulated by apob RNAi.

Supplementary Data 6. Gene and transcript identities used in phylogenetic analyses and gene expression studies.

Supplementary Data 7. RNA-Seq Data from NCBI GEO GSE 107874 mapped to the dd_Smed_v6 transcriptome.
