# Supplementary material for: Intestine-enriched apolipoprotein b orthologs are required for stem cell progeny differentiation and regeneration in planarians
Source: Nat Commun. 2022 Jul 1;13:3803. doi: 10.1038/s41467-022-31385-2 (PMC9249923; doi:10.1038/s41467-022-31385-2)
Supplement: Supplementary file 11 — Reporting Summary [file 41467_2022_31385_MOESM11_ESM.pdf]

nature research

Corresponding author(s): David J. Forsthoefel, Ph.D.

Last updated by author(s): 05/31/2022

Reporting Summary

Nature Research wishes to improve the reproducibility of the work that we publish. This form provides structure for consistency and transparency in reporting. For further information on Nature Research policies, see our Editorial Policies and the Editorial Policy Checklist.

Statistics

For all statistical analyses, confirm that the following items are present in the figure legend, table legend, main text, or Methods section.

n/a	Confirmed	
☐	☒	The exact sample size (*n*) for each experimental group/condition, given as a discrete number and unit of measurement
☐	☒	A statement on whether measurements were taken from distinct samples or whether the same sample was measured repeatedly
☐	☒	The statistical test(s) used AND whether they are one- or two-sided<br>*Only common tests should be described solely by name; describe more complex techniques in the Methods section.*
☒	☐	A description of all covariates tested
☐	☒	A description of any assumptions or corrections, such as tests of normality and adjustment for multiple comparisons
☐	☒	A full description of the statistical parameters including central tendency (e.g. means) or other basic estimates (e.g. regression coefficient) AND variation (e.g. standard deviation) or associated estimates of uncertainty (e.g. confidence intervals)
☐	☒	For null hypothesis testing, the test statistic (e.g. *F*, *t*, *r*) with confidence intervals, effect sizes, degrees of freedom and *P* value noted<br>*Give P values as exact values whenever suitable.*
☒	☐	For Bayesian analysis, information on the choice of priors and Markov chain Monte Carlo settings
☐	☒	For hierarchical and complex designs, identification of the appropriate level for tests and full reporting of outcomes
☒	☐	Estimates of effect sizes (e.g. Cohen's *d*, Pearson's *r*), indicating how they were calculated

Our web collection on statistics for biologists contains articles on many of the points above.

Software and code

Policy information about availability of computer code

Data collection	Protein identification: BLAST (2.6.0 locally; later versions at blast.ncbi.nlm.nih.gov/Blast.cgi), HMMSCAN (https://www.ebi.ac.uk/Tools/hmmer/search/hmmscan, v.2.29+ at HMMER web server). Western analysis: Wes Instrument (running Compass v4.0.0). TLC: Alpha Innotech chemiluminescent imager. qPCR: Roche LightCycler 96 (SW 1.01.01.0050). Flow cytometry: BD FACSDiva (v9.0). Microscopy: Zeiss AxioObserver.Z1 (ZEN 2.3 Blue); Zeiss LSM 710 (ZEN version 11.0.3.190, 2012-SP2); Zeiss LSM 880 (ZEN Black 2.3 SP1 FP3); Zeiss Stemi 508 (ZEN 2.3 Blue); Zeiss AxioZoom.V16 (ZEN 2012 Blue).
Data analysis	Protein alignment and phylogenetic analysis: Geneious Prime 2020.2.3 (MAAFT v7.450), PhyML 3.0 (http://www.atgc-montpellier.fr/phyml/). Image analysis: ImageJ/Fiji (2.3.0/1.53f , including the Phansalkar Method in Auto Local Threshold v1.10.1), Zeiss ZEN (multiple versions, above), Adobe Photoshop. Wes analysis: Compass v5.0.1 for Mac. qPCR: Roche LightCycler 96 SW 1.1 and Microsoft Excel. RNA-Seq and bioinformatics: FastQC 0.11.5, BBDuk 35.66, Bowtie2 2.3.1, Subread 1.6.3, Samtools (multiple versions), R 3.3.0-3.6.0, edgeR 3.26.3 (apob (RNAi) RNA-Seq); edgeR 3.16.5 (nkx2.2(RNAi) RNA-Seq and Zeng et al. RNA-Seq datasets), Cytoscape 3.8.0, BiNGO 3.0.4, Cluster 3.0, Java TreeView 1.1.6r4 and later). Flow cytometry: FlowJo 10.7.1. Statistics: Prism 9, GeneOverlap 1.20.0. Custom code for image analysis and cross-referencing of bulk and single-cell RNA-Seq data have been deposited in Zenodo.org (https://doi.org/10.5281/zenodo.6596520 and https://doi.org/10.5281/zenodo.6596518).

For manuscripts utilizing custom algorithms or software that are central to the research but not yet described in published literature, software must be made available to editors and reviewers. We strongly encourage code deposition in a community repository (e.g. GitHub). See the Nature Research guidelines for submitting code & software for further information.

Data

Policy information about availability of data

All manuscripts must include a data availability statement. This statement should provide the following information, where applicable:

- Accession codes, unique identifiers, or web links for publicly available datasets
- A list of figures that have associated raw data
- A description of any restrictions on data availability

Raw and processed RNA-Seq data associated with this study are available in the NCBI Gene Expression Omnibus (GEO) under records GSE174246, GSE174227, and GSE174228 (Supplementary Data 1 & 5). Differential expression analysis of RNA-Seq reads from NCBI GEO GSE 107874 mapped to the dd_Smed_v6 transcriptome are provided in Supplementary Data 7. Other data supporting this study's findings are available within the article and its Supplementary files, or are available from the authors upon reasonable request. Source data are provided with this paper.

Field-specific reporting

Please select the one below that is the best fit for your research. If you are not sure, read the appropriate sections before making your selection.

☒ Life sciences ☐ Behavioural & social sciences ☐ Ecological, evolutionary & environmental sciences

For a reference copy of the document with all sections, see nature.com/documents/nr-reporting-summary-flat.pdf

Life sciences study design

All studies must disclose on these points even when the disclosure is negative.

Sample size	No power analysis or other statistical methods were used to determine sample sizes. However, number of biological replicates/animals scored per assay were chosen based on similar previously published experiments. Examples: WISH/FISH (Hill and Petersen, Development, 2015; Lander and Petersen, eLife, 2016); anti-phosphoHistoneH3-S10 labeling (Lei et al., Dev. Cell, 2017; Lin et al., PLoS Genetics, 2017); brain size quantification (Roberts-Galbraith et al., eLife, 2016); pharynx size quantification (Adler et al., eLife, 2014); epidermal cell counting (van Wolfswinkel et al., Cell Stem Cell, 2014); flow cytometry (Berrett et al., Immunohorizons, 2019); thin layer chromatography (Rambold et al., Dev. Cell, 2015); qPCR (Owen et al., Development, 2015); section quantification (Scimone et al., Current Biology, 2018); Oil Red O labeling (Zhang et al., bioRxiv, 2018); RNA-Seq (Cowles et al., PLoS Genetics, 2014). For all experiments, sample sizes are listed explicitly in Figure Legends and/or Source Data.
Data exclusions	No data were excluded from analysis, except for damaged specimens for which complete imaging or quantification was not possible. In RNA-Seq experiments, reads and transcripts were filtered based on quality and expression levels as described in Methods.
Replication	For most quantitative experiments, results are representative of at least two independent studies, indicated in Figure Legends. Experiments in Fig. 4h-i, Supp. Fig. 4c, and Fig. 6a-g were performed once due to time constraints, and because results from multiple flow cytometry experiments with anti-PIWI-1 labeling (Fig. 6h-k) independently supported conclusions drawn from these results. In micrographs displaying gene expression without quantification (e.g., Fig. 1a-b, Supp. Fig. 1a, Supp. Fig. 2f-g, Supp. Fig. 3b and 3d, Supp. Fig. 4a-b) experiments were performed once, but with similar results in most biological replicates (animals/sections), indicated in Figure Legends. Experiments in Supp. Fig. 2d-e were not repeated because results from independent experiments in Fig. 2d-e supported similar conclusions. RNA-Seq experiments were performed once due to resource constraints, but with 3-6 biological replicates per condition (details in Methods) that were similar to each other (shown in Supp. Fig. 5c for control vs. apob(RNAi) samples and provided as Supp. Fig. 1b Data in Source Data for control vs. nkx2.2(RNAi) samples).
Randomization	For all experiments, animals were randomly selected from large (300-500 animals) pools, and randomly placed into experimental groups. For selection of apob “mild” and “severe” samples, assignment to each category was verified by one additional researcher before animals were randomly placed into experimental groups.
Blinding	Investigators were not blinded during data acquisition or analysis. However, qualitative data (Fig. 1, Fig. 2a/c/d, Fig. 3b, Supp. Fig. 1a/g/h, Supp. Fig. 2d-g, Supp. Fig. 3b/d, Supp. Fig. 4a-b) and quantitative in vivo/in situ data (Fig. 3c-h, Fig. 4a-b, Fig. 6g) were evaluated independently by at least one additional researcher. For automated image analysis, flow cytometry, and RNA-Seq, identical instrument settings and software parameters were utilized for quantification and analysis of all biological and technical groups.

Reporting for specific materials, systems and methods

We require information from authors about some types of materials, experimental systems and methods used in many studies. Here, indicate whether each material, system or method listed is relevant to your study. If you are not sure if a list item applies to your research, read the appropriate section before selecting a response.

Materials & experimental systems

n/a	Involved in the study
☐	☒ Antibodies
☒	☐ Eukaryotic cell lines
☒	☐ Palaeontology and archaeology
☐	☒ Animals and other organisms
☒	☐ Human research participants
☒	☐ Clinical data
☒	☐ Dual use research of concern

Methods

n/a	Involved in the study
☒	☐ ChIP-seq
☐	☒ Flow cytometry
☒	☐ MRI-based neuroimaging

Antibodies

Antibodies used

anti-ApoB-1 (custom S. mediterranea anti-ApoB-1 generated for this study by GenScript USA - Piscataway, NJ)
anti-PIWI-1 (custom S. mediterranea anti-planarian PIWI-1 generated for this study by GenScript USA - Piscataway, NJ)
anti-DIG-POD (Roche 11207733910)
anti-DIG-AP (Roche 11093274910)
anti-FITC-POD (Roche 11426346910)
anti-DNP-HRP (Perkin Elmer FP1129 /Akoya Biosciences TS-000400)
anti-muscle 6G10 (Developmental Studies Hybridoma bank 6G10-2C7)
anti-H3P (Rabbit, Cell Signaling 3377S)
normal rabbit IgG (Jackson ImmunoResearch 011-000-003)
anti-rabbit-HRP (Jackson ImmunoResearch 111-035-003)
anti-rabbit-HRP (Jackson ImmunoResearch 111-035-144)
anti-rabbit-488 (Jackson ImmunoResearch 111-545-144)
anti-mouse-488 (Jackson ImmunoResearch 115-545-146)
anti-rabbit-647 (Jackson ImmunoResearch 711-605-152)

Validation

Validation of custom anti-ApoB-1 (generated against S. med. ApoB-1 for this study) was performed by comparing signals from control and apob(RNAi) animals, using Western analysis on protein extracts and immunofluorescence on tissue sections (details in Figure 2, Methods, and Source Data). Signal was reduced ~90% by Western analysis in apob(RNAi) animals. Validation of anti-PIWI-1 (generated against an S. med. peptide sequence for this study) was performed by comparing signals in lethally irradiated and un-irradiated samples one day post irradiation by flow cytometry of dissociated planarian cells (details in Supplementary Figure 10 and Methods). >90% of PIWI-1-high cells were eliminated in 24 hr post-irradiated animals, consistent with previously published work (Guo et al., Dev. Cell, 2006 and Zeng et al., Cell, 2018). Other primary antibodies have been extensively validated by published literature in the planarian field (e.g., 6G10 - Ross et al., BMC Dev. Biol., 2015; anti-H3P - Forsthoefel et al., Methods Mol. Biol., 2018 and Barberán et al., Development, 2016).

Animals and other organisms

Policy information about studies involving animals; ARRIVE guidelines recommended for reporting animal research

Laboratory animals

Asexual Schmidtea mediterranea planarians (clonal line CIW4) (Sánchez Alvarado et al., Development, 2002) were maintained under standard laboratory conditions. Asexual S. mediterranea are neither male nor female, reproduce asexually by fissioning, and under normal laboratory conditions are either very long-lived or immortal (Vila-Farré and Rink, Methods Mol. Biol., 2018). Therefore sex and age are not currently believed to be a significant source of experimental variability, and are not distinguished during selection of animals for experiments.

Wild animals

No wild animals were used in this study.

Field-collected samples

No field-collected organisms were used in this study.

Ethics oversight

For anti-ApoB1 and anti-PIWI-1 generation, animal welfare protocols of GenScript USA (an OLAW, AAALAC, and PHS-approved vendor) were approved by OMRF IACUC (17-58).

Note that full information on the approval of the study protocol must also be provided in the manuscript.

Flow Cytometry

Plots

Confirm that:

- [x] The axis labels state the marker and fluorochrome used (e.g. CD4-FITC).
- [x] The axis scales are clearly visible. Include numbers along axes only for bottom left plot of group (a 'group' is an analysis of identical markers).
- [x] All plots are contour plots with outliers or pseudocolor plots.
- [x] A numerical value for number of cells or percentage (with statistics) is provided.

Methodology

Sample preparation	Planarians were dissociated in CMFB with 1mg/ml collagenase. Cells were resuspended in CMFB and were labeled at RT with Hoechst 33342 (50 ug/ml) for 45 min, followed by addition of propidium iodide (1 ug/ml). For neutral lipid labeling, BODIPY 493/503 (Molecular Probes D3922) at 10 ng/ml was included with Hoechst. For anti-PIWI-1 labeling, dissociated cells were fixed in 2% para-formaldehyde, permeabilized and blocked for 30 min before incubation with anti-PIWI-1 (0.2 ug/ml) overnight at 4C, and anti-rabbit-Alexa-488 (1:8000) for 30 min.
Instrument	Becton Dickinson FACSCelesta
Software	Acquisition: BD FACSDIVA software v9.0. Analysis: FlowJo v10.7.1.
Cell population abundance	X1, X2, and Xins populations were present at 10-65% of all propidium iodide (PI) negative and Hoechst positive cells, except in irradiated samples, in which X1 population was 0.2-1.5% of all cells. PIWI-HI and PIWI-LO populations were present at 10 to 40%, except in irradiated samples, in which PIWI-HI was ~1%.
Gating strategy	Particles were first gated on FSC-H/FSC-A events to eliminate doublets, then PI negative events, then Hoechst positive cells then X1, X2, and Xins subpopulations as indicated in Supplementary Fig. 11a-c and Fig. 2g, h. For anti-PIWI-1 labeling, particles were first gated on FSC-H/FSC-A events to eliminate doublets, then DAPI positive cells, then SSC against PIWI-1 (AF488) to segregate PIWI-LO and PIWI-HI populations. See Supplementary Fig. 11d-f for examples.

- [x] Tick this box to confirm that a figure exemplifying the gating strategy is provided in the Supplementary Information.
